# Towards One-Step Quantitation of Prostate-Specific Antigen (PSA) in Microfluidic Devices: Feasibility of Optical Detection with Nanoparticle Labels

**DOI:** 10.1007/s12668-016-0390-y

**Published:** 2017-01-07

**Authors:** Ana I. Barbosa, Jan H. Wichers, Aart van Amerongen, Nuno M. Reis

**Affiliations:** 1 Department of Chemical Engineering, Loughborough University, Loughborough, LE11 3TU UK; 2 BioSensing and Diagnostics, Wageningen University and Research, Bornse Weilanden 9, 6708 WG Wageningen, The Netherlands; 3 Department of Chemical Engineering, University of Bath, Bath, BA2 7AY UK

**Keywords:** Carbon nanoparticles, Gold nanoparticles, Microfluidics, Microcapillary film, Prostate-specific antigen

## Abstract

Rapid and quantitative prostate-specific antigen (PSA) biomarker detection would be beneficial to cancer diagnostics, improving early detection and therefore increasing chances of survival. Nanoparticle-based detection is routinely used in one-step nitrocellulose-based lateral flow (LF) immunoassays; however, it is well established within the scientific diagnostic community that LF technology lacks sensitivity for measuring biomarkers, such as prostate-specific antigen (PSA). A trend in point-of-care (POC) protein biomarker quantitation is the miniaturization of immunoassays in microfluidic devices. This work aimed at testing the feasibility of carbon and gold nanoparticles as immunoassay labels for PSA detection with cost-effective optical detection in a novel microfluidic POC platform called microcapillary film (MCF), consisting of a parallel array of fluoropolymer microcapillaries with 200-μm internal diameter. With neutravidin-coated carbon, nanoparticles were able to quantify an immobilized biotinylated monoclonal antibody (coating solution from 10 to 40 μg/ml) and PSA was successfully quantified in a sandwich assay using silver-enhanced gold nanoparticles and a flatbed scanner; yet, the dynamic range was limited to 10–100 ng/ml. Although direct optical detection of PSA without enzymatic amplification or fluorophores is possible and technically appealing for the simplified fluidics and signal scanning setups involved, ultimately, the binding of a thin layer of nanoparticles onto the wall of transparent microcapillaries is not sufficient to cause a significant drop on the optical colorimetric signal. Future studies will explore the use of fluorescence nanoparticles.

## Introduction

A point-of-care (POC) test has to be sensitive, capable of detecting a broader number of clinical conditions, and the simplicity of use should approach that of a nitrocellulose lateral flow test (LF). Heterogeneous immunoassays, where the free analyte is separated from the bound forming the antibody-antigen complex, are the most sensitive bioanalytical tools used in clinical diagnostics. However, when translated to POC format, the sensitivity of the test is often compromised by the required level of simplicity and speed of the assay, in addition to the need of using small volumes of complex biological matrices (sample) and low-cost optical scanning [1].

In most heterogeneous sandwich immunoassays, the antibody-antigen complex is quantified through label detection. The label conjugated to a detection antibody (DetAb) generates a signal which is proportional to the concentration of antigen. The nature of signal depends on the label used which in turn defines the readout system to be used. These two aspects work together establishing the overall sensitivity of the assay.

The first labels used in immunoassays were radioactive isotopes, such as iodine-125 [2]. However, due to concerns about radioactivity exposure, disposal of radioactive waste and instability of radiolabels reagents, other labels were developed. [3] Enzymes [4], fluorophores [5], chemiluminophores [6], microparticles [7] and quantum dots [8] have been extensively applied to signal generation in immunoassay tests including miniaturized microfluidic devices. Enzymes are the most widely used label due to their flexibility and unique amplification power. Different enzymes are available that can generate coloured, fluorescent or luminescent products from a transparent substrate. Enzymes are strong signal amplifiers, which are established by the turnover number. A single enzyme molecule can convert up to 10^7^ molecules of substrate per minute [2]. However, enzymes are not suitable for one-step immunoassays, as they require more complex washings and the use of multiple reagents.

LF immunoassays are a well-established technology for one-step POC testing, and current applications include detection of pregnancy hormone (hCG), malaria and HIV. Nevertheless, LF technology lacks sensitivity which disables it to diagnose certain clinical conditions that demand sensitive quantitation of biomarkers. This is the case with different types of cancer biomarkers including prostate-specific antigen (PSA), which presents a clinical threshold of 4 ng/ml for biopsy [9] and 0.4 ng/ml for monitoring cancer recurrence after radical prostatectomy [10–14]. The POC detection of protein biomarkers like PSA is in need of simple and rapid tests that preferably use a drop of blood collected from a finger prick or from an intravenous blood sample or other biological sample (e.g. urine) and a very simple fluidic setup, ideally as one-step immunoassay [1, 15]. Microfluidic tests qualify to fill up this gap since they are able to precisely control the flow and use a broader range of sample volumes. However, translating LF assays into microfluidic platforms poses several technical challenges.

LF traditionally uses a label directly conjugated to the DetAb without the need of signal amplification. The sample is placed on a sample pad, moving through by capillary action, mixes with the labelled antibody in the conjugated pad, and continues to flow through the test zone [16]. The density of labelled particles in the test zone generates a signal proportional to the analyte concentration. LF technology uses porous membranes, such as nitrocellulose, which present a very large surface area even in a very small test zone (1-mm width line). Translating one-step immunoassays into microfluidic platforms with a much lower order of magnitude of surface area in the detection zone can be challenging, probably compromising the sensitivity of the test.

Traditionally, particles have been used as labels in microfluidic systems, yet the readout system has to be sophisticated or amplification methods have to be applied [17, 18]. To combine good sensitivities with multiplex capability, microfluidic systems often use fluorophores as labels, which cannot be considered particles. Despite this, signal detection from fluorescent labels requires the use of complex and expensive equipment, such as fluorescent microscopes, flow cytometers and fluorescent scanners [5, 19].

Few examples of microfluidic immunoassays can be found in literature which used particles as labels and low-cost readout systems. Yet C. et al. (2009) [20] reported a protein A sandwich immunoassay where the capture antibody (CapAb) was immobilized onto glass slides, surrounded by a polydimethylsiloxane (PDMS) structure. The DetAb, conjugated with platinum nanoparticles, generated a signal which was subsequently enforced by a silver enhancement step. The signal was detected by a flatbed scanner with a reported limit of detection (LoD) of the assay in the order of 1 ng/ml [20]. Lu Y. et al. (2009) [21] reported a human IgG/anti-IgG assay, with IgG immobilized onto the polystyrene surface inside PDMS channels, with the DetAb labelled with gold nanoparticles and also silver enhanced. The test was optically scanned with a cell phone camera, with a LoD of approximately 1 ng/ml [21]. PSA presented a LoD of 5 × 10^−4^ pM with a biobarcode using the Verigene ID scanning system. [22] Understanding how particle labels can be detected with low-cost readout systems can be transformative to microfluidic immunoassays, bringing them closer to an ideal POC diagnostic test.

Recently, PSA has been quantified using a ‘lab-in-a-briefcase’ and portable smartphone detection with colorimetric and fluorescent multistep enzymatic amplification achieving a LoD of 0.08 to 0.9 ng/ml [23, 24]. In these assays, the detected signal related to the whole capillary volume occupied by the converted chromogenic or fluorescent substrate, which was optically scanned with low-cost detection readers. This present work aims reducing the number of assay steps by eliminating the enzymatic reaction and performing direct detection of nanoparticle labels adsorbed on the capillary wall.

**Fig. 1:**
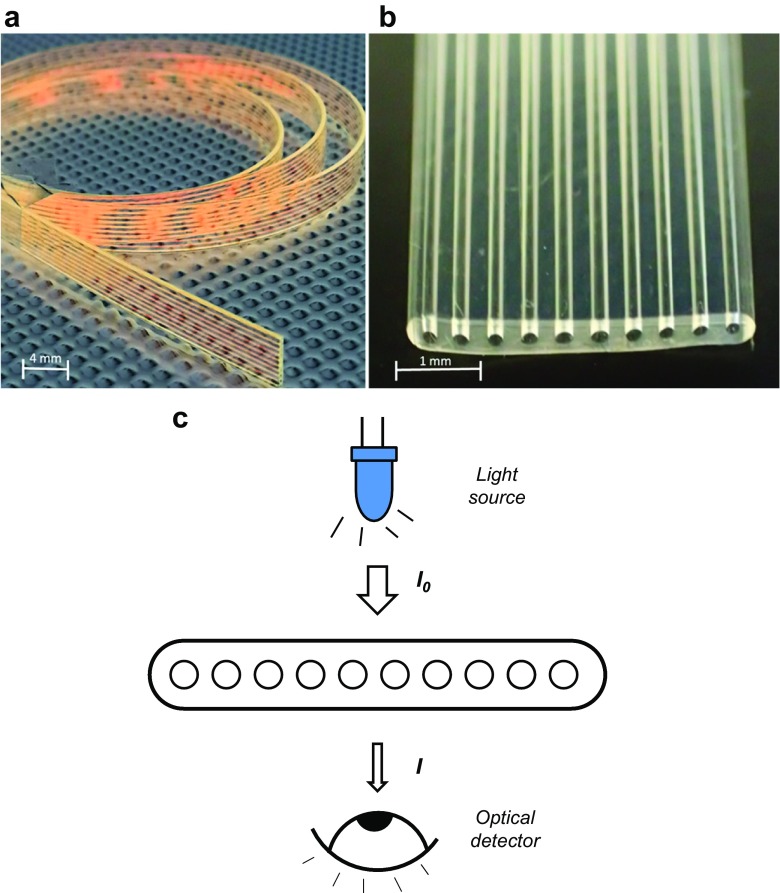
Microcapillary film (MCF) immunoassay platform. **a** Close look of MCF. **b** Microphotograph of MCF showing the transparency of the FEP-Teflon polymer and the 10 parallel capillaries with 200-μm diameter average. **c** Optical interrogation of MCF immunoassay test strips

**Fig. 2:**
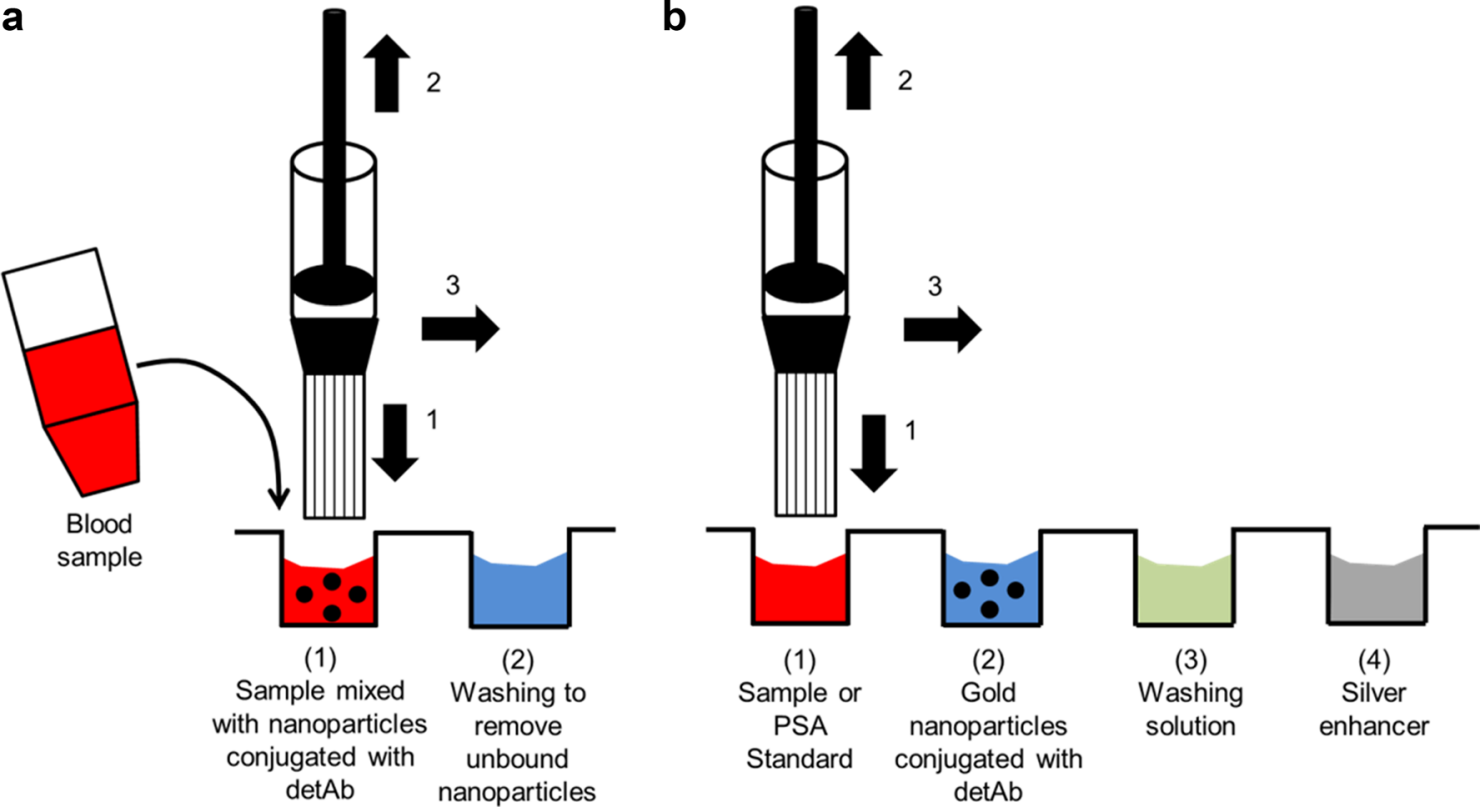
Nanoparticle-labelled assay methodology in the MCF platform. The MCF is attached to a syringe and inserted into a well with a reagent solution (*1*), 200 μl of that solution is aspirated (*2*) and the reagent will be aspirated from the adjacent well (*3*). **a** Our vision for a simple two-step MCF assay. **b** Assay methodology performed in the present experimental work, described in Section 2.3

This paper reports for the first time the use of two different particle labels, carbon nanoparticles [25] and gold nanoparticles for optical quantitation of PSA in transparent plastic microcapillary film (MCF, Fig. 1). Carbon nanoparticles have been reported to be 100 and 10 times more sensitive than standard gold nanoparticles and silver enhanced gold nanoparticles, respectively. For this reason, both types of nanoparticles were used in this study in order to find the optimum label for PSA detection [26]. The binding of nanoparticles to the inner walls of the microcapillaries pre-coated with CapAb causes an optical diffraction, resulting in a drop of the optical colorimetric signal. Optical nanoparticle detection of PSA and other biomarkers in microcapillaries can potentially help reducing the assay time and complexity of the overall assay, bringing these microfluidic tests closer to a POC format (Fig. 2a). The MCF is a melt-extruded fluorinated ethylene propylene (FEP) -Teflon flat film, with 10 parallel microcapillaries with 200 μm mean internal diameter. This geometry offers a similar surface area as a microwell in a 96-well plate, considering a 1-cm long MCF strip, but a surface-area-to-volume ratio that is 15 to 20-fold higher.

## Materials and Methods

### Materials and Reagents

Purified anti-human IL-1β biotin and anti-human IL-1β were supplied by e-Biosciences (Hatfield, UK). Anti-PSA CapAb and anti-PSA DetAb conjugated with gold nanoparticles were supplied by Wama Diagnóstica (São Carlos, Brazil). The silver enhancement kit was supplied by Sigma-Aldrich (Dorset, UK). The PSA native protein was purchased from Abcam (Cambridge, UK). The Carbon nanoparticles were supplied by Wageningen Food and Biobased Research, Wageningen University & Research (Wageningen, The Netherlands). The particles cluster to irregular shapes with an average size of 150 to 200 nm.

Phosphate-buffered saline (PBS) and bovine serum albumin (BSA) were sourced from Sigma-Aldrich (Dorset, UK). PBS pH 7.4, 10 mM was used as the main experimental buffer. The blocking solution consisted of 3% *w*/*v* protease-free BSA diluted in PBS buffer. For washings, PBS with 0.05% *v*/*v* of Tween-20 (Sigma-Aldrich, Dorset, UK) was used. The FEP-Teflon microcapillary film (MCF) is fabricated from fluorinated ethylene propylene co-polymer by melt-extrusion process by Lamina Dielectrics Ltd. (Billingshurst, West Sussex, UK). FEP-Teflon MCF presents 10 bore parallel capillaries with 223 ± 23-μm diameter.

### Neutravidin-Coated Carbon Nanoparticle Detection

Four solutions of IL-1β biotinylated with 0, 10, 20, 40 and 80 μg/ml and one with 3% of BSA were injected in different capillaries using a 1-ml syringe attached with a needle (i.d. 200 μm) of a 24-cm MCF strip and incubated for 2 h at room temperature. The biotinylated antibody solution was replaced by 3% BSA solution incubated for 2 h at room temperature in all the capillaries. The long MCF strip was trimmed in 4-cm length strips and several dilutions of neutravidin conjugated carbon nanoparticle solutions (1:1, 1:2, 1:5, 1:7, 1:10 and 1:50) were added to the different 4-cm MCF strips being incubated for 2 h in an orbital shaker, followed by a washing step with PBS Tween. The strips were then imaged with a flatbed scanner (HP Scanjet G4050) in transmittance mode.

The MCF was cut exposing the surface area coated with the carbon nanoparticles, gold-coated for 5 min with a gold sputter cater/carbon evaporator and placed in a high-resolution field emission gun scanning electron microscope (FEGSEM) for imaging at several magnifications, being the highest is 15,000 KW.

### Gold Nanoparticle Detection with Silver Enhancement

An MCF strip was filled with 40 μg/ml of anti-PSA CapAb and incubated for 2 h at room temperature. The solution was replaced by 3% BSA incubated for 2 h at room temperature. After a washing step, the strip was trimmed in 4-cm MCF test strips. Four MCF test strips were filled with 0, 10, 50 and 100 ng/ml of PSA native protein solutions and incubated for 10 min. A solution of 1:50 dilution of anti-PSA DetAb conjugated with gold nanoparticles (Fig. 2b) was incubated for 1 h in the orbital shaker followed by a washing step. The silver enhancement solution was applied for 10 min at room temperature and the fixing solution for 3 min followed by a distilled water washing step. The MCF strips were imaged with the flatbed scanner in transmittance mode (Fig. 1c).

### MCF Image Analysis

RGB digital images were split into 3 separate channel images by ImageJ software. The green channel images were used to calculate absorbance values, based on the grey scale peak height of each individual capillary of FEP-Teflon® MCF as described previously [27, 28] and briefly illustrated in Fig. 1c. The absorbance value on each individual microcapillary was calculated based on Eq. (1):

1 $$ \mathrm{Abs}=\hbox{-} \log \left(\frac{I}{I_0}\right) $$

where *I* is the grey scale peak height and *I*_0_ is the maximum grey scale value. The absorbance values presented averages of absorbance from 10 capillaries to one MCF stip.

## Results and Discussion

### The Use of Carbon Nanoparticles

The use of particles as labels instead of enzymes can bring microfluidic systems closer to simple one-step POC diagnostics, eliminating the time and complexity of multiple step immunoassays. Only few studies have been reported dealing with particle detection in microfluidic systems with low-cost detection systems. However, such systems are fundamental for future development of affordable POC biomarker testing.

**Fig. 3:**
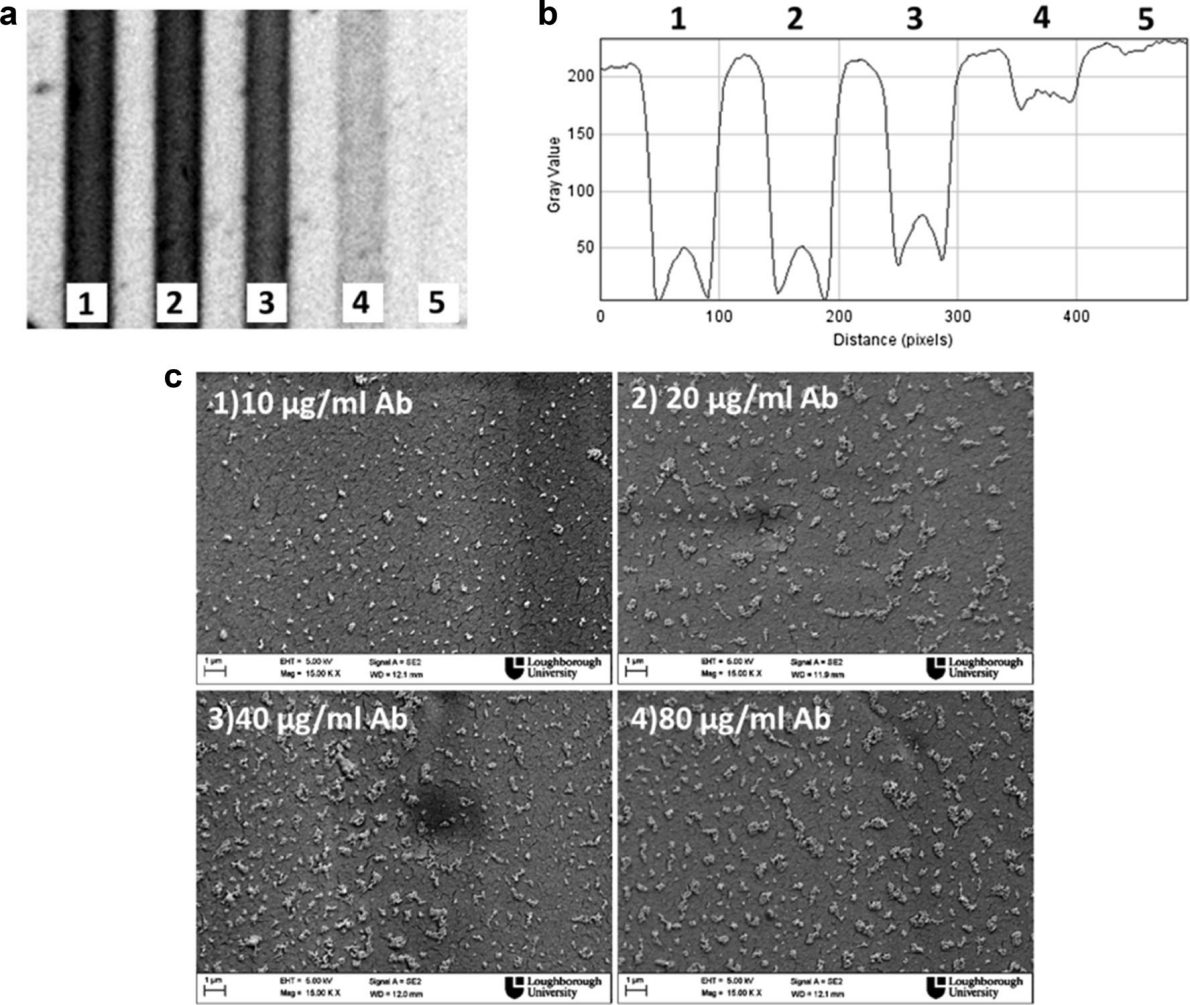
Biotinylated antibody MCF-adsorbed detection with carbon nanoparticles neutravidin coated (1:10 dilution). **a** MCF flatbed scanner green channel digital image in transmittance mode, showing microcapillaries coated with 80, 40, 20, 10 and 0 μg/ml of biotinylated antibody, 1 to 5, respectively. **b** Corresponding grey scale showing the concentrations of 80, 40, 20, 10 and 0 μg/ml of biotinylated antibody, 1 to 5, respectively. **c** Scanning electron microscope (SEM) pictures with 10, 20, 40 and 80 μg/ml of biotinylated antibody

Carbon nanoparticles were used in LF assays for albumin detection with a LoD of 0.25 μg/ml, and Kunitz-type for trypsin inhibitor was detected with a LoD of 2.5 ng/ml in competitive assay format [29]. A study that aimed to enhance the detection limit of LF test through the evaluation and comparison of bioconjugates reported a 10-fold improvement in sensitivity for biotin-streptavidin systems and for dengue detection when compared with silver-coated gold nanoparticles [26]. Carbon nanoparticles coated with neutravidin were the first carbon particle system to be tested in the MCF platform. Particles bound directly to immobilize biotinylated antibodies and a dynamic range between 10 and 40 μg/ml of the biotin-antibody coating solution was observed by flatbed scanner. Digital images were collected in transmittance mode and followed by grey scale analysis (Fig. 3a, b). In the grey scale image (Fig. 3b), it is possible to score the difference in the refractive index of MCF capillary walls with and without particles attached.

**Fig. 4:**
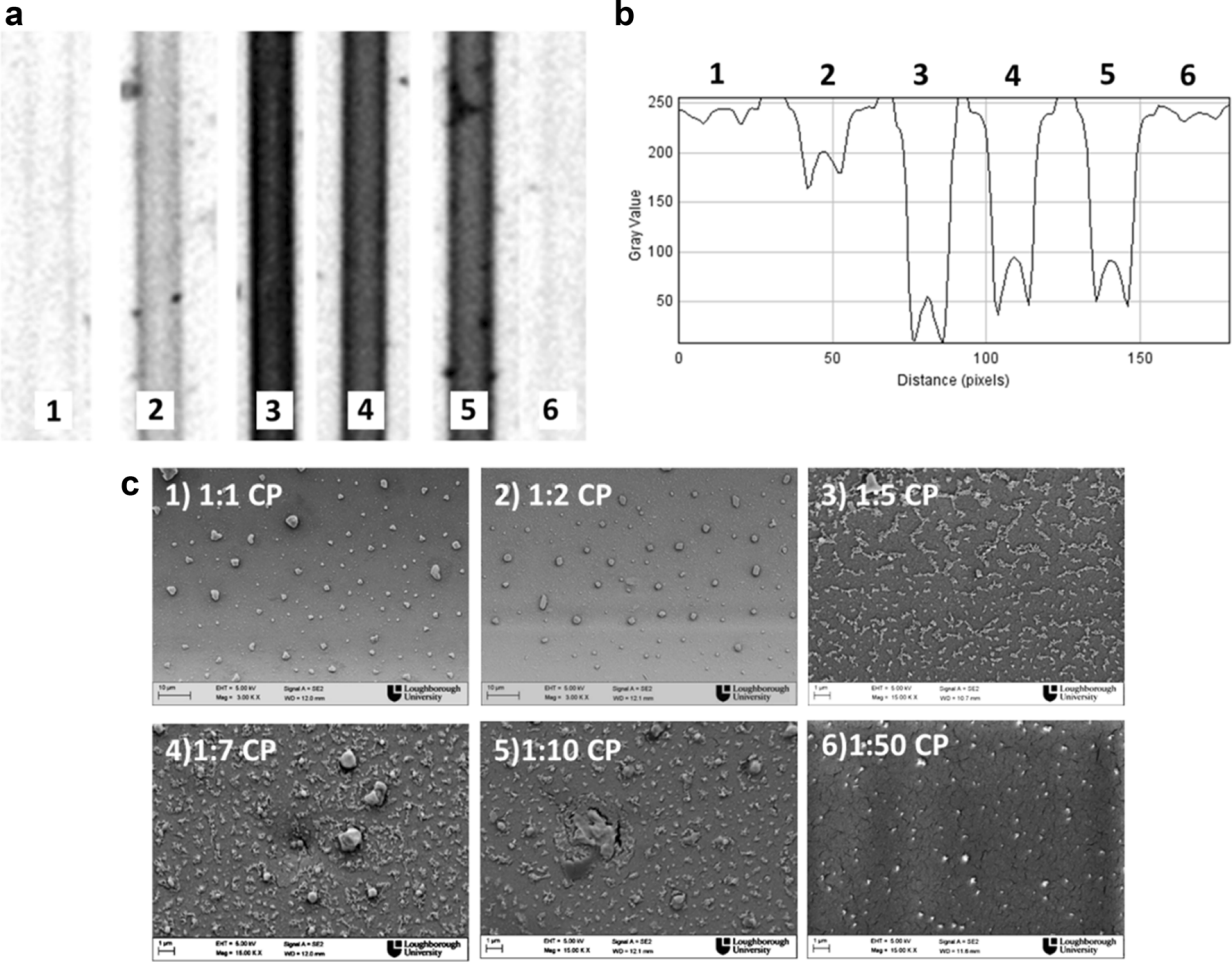
Carbon nanoparticle neutravidin-coated detection of 40 μg/ml of biotin-antibody in the MCF. **a** Flatbed scanner digital image of microcapillaries with 40 μg/ml of biotin-antibody and several dilutions of carbon nanoparticles 1, 2, 3, 4, 5 and 6 which correspond to 1:1, 1:2, 1:5, 1:7, 1:10 and 1:50, respectively. **b** Correspondent grey scale image with previously mentioned dilutions of carbon nanoparticles and 40 μg/ml of biotin-antibody. **c** Correspondent scanning electron microscope (SEM) pictures with several dilutions of carbon nanoparticles

The SEM images in Fig. 3 are coherent with the flatbed scanner images showing an increment in the surface density of carbon nanoparticles from 10 μg/ml and moving towards saturation beyond 40–80 μg/ml biotin-antibody in the coating solution. This showed that it is possible to detect carbon nanoparticles using a flatbed scanner as a readout system, however requiring further optimization. Potentially, other detection systems should be tested in order to achieve better sensitivity and apply this nanoparticle label to sandwich immunoassays. Optimization started with screening the concentration of carbon nanoparticles and the results are shown in Fig. 4.

High concentrations of carbon nanoparticles were not beneficial for molecular detection in the MCF as shown in Fig. 4, as flatbed scanner images with a coating concentration of 40 μg/ml of biotinylated antibody showed no signal with undiluted or even 1:2 diluted carbon nanoparticles. In the SEM images of microcapillaries (Fig. 4c), it is possible to see crystals with an average perimeter size of 3 μm. Considering the carbon particles are irregular in shape and present an average size of 150 to 200 nm, we conclude that in certain conditions, the carbon nanoparticles agglomerated, becoming larger and more prone to be removed during the washing steps. Carbon nanoparticles diluted above 1:50 are not recommended either, as there is not enough surface density of particles to cause a decrease in optical signal that is detected with the flatbed scanner, despite the nanoparticles being clearly visible in the SEM images (Fig. 4c). As can be seen in Fig. 4b, higher signals were obtained with the carbon dilutions of 1:5 to 1:10, dilution 1:5 giving the best results.

Optimizing the carbon particle concentration would not be enough to perform sensitive immunoassay in the MCF, as the maximum surface density achieved is far from full surface coverage and the total surface area available is not large enough to promote an optical signal that can be detected e.g. with naked eye. Thus, a full PSA sandwich immunoassay in MCF with carbon nanoparticles was not attempted in this study. Using different light sources and optical setup with a portable camera could improve signal intensity of nanoparticles in microcapillaries.

### The Use of Gold Nanoparticle Label with Silver Enhancement

**Fig. 5:**
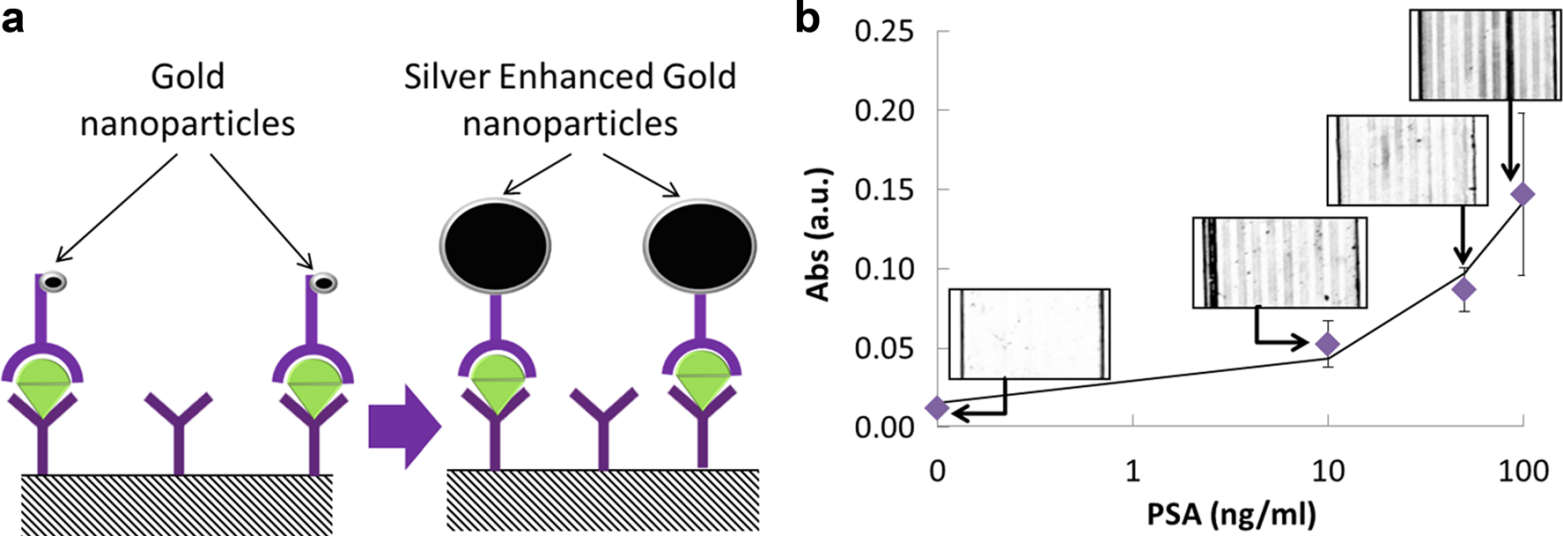
Gold nanoparticle MCF IAs with silver enhancement. **a** Sandwich assay diagram with gold nanoparticle silver enhancement step. **b** PSA response curve with gold nanoparticle silver enhanced; *inset* pictures show scanned images of the MCF strips

The other nanoparticle system tested in the MCF platform for PSA detection was the silver-enhanced gold nanoparticles. A PSA sandwich assay was performed, and the results are shown in Fig. 5. PSA is a prostate cancer biomarker with a clinical threshold of 4 ng/ml [9]. The data obtained showed a significant difference between 0 ng/ml of PSA and the lowest concentration of PSA tested, which was 10 ng/ml. This indicates that the sensitivity of the assay may be improved upon optimization of the assay. A 4PL (4 Parameters Logistic) model fitted the data with a cross correlation coefficient of 0.999. However, the variability between capillaries of the same MCF test strip is 34% in the highest PSA concentration. This shows that the concentration of gold nanoparticles might not be optimized yielding poor uniformity for higher PSA concentrations, since there are not enough gold nanoparticles to bind to all the antigens immobilized in the 10 capillaries. Alternative optical setups will also be explored in the future for enhancing signal-to-noise ratio.

To the best of our knowledge, this is the first time a cancer biomarker (PSA in this case) is quantified using the silver-enhanced gold nanoparticles in a microfluidic platform. Although the silver enhancement results in a multistep assay, particle labels are flexible, cost effective and will, therefore, contribute to increase flexibility of microfluidic POC systems.

### Limitations of Optical Detection with Nanoparticle Labels

**Fig. 6:**
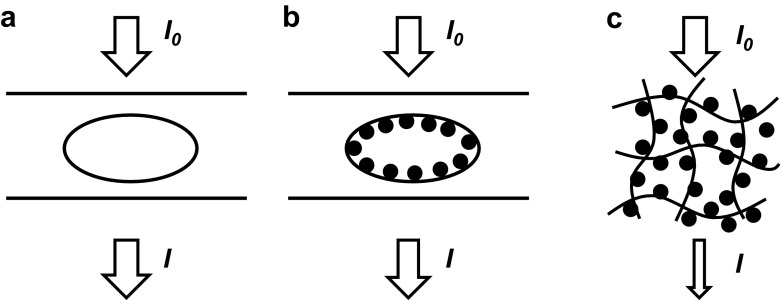
Optical interrogation of heterogeneous immunoassay tests. **a** In the absence of any optical signal (particles or enzymatic), the transmitted light equals the incident light, resulting in zero absorbance value. **b** With the use of colloidal carbon or gold nanoparticles, particles bound to the inner wall of the microcapillary lead to a change of the refractive and reduction on the transmitted light; however, strength of optical signal is limited by the monolayer. **c** In a porous media like a nitrocellulose membrane, the larger surface area available allows stronger optical signals to be generated (transmitted or reflectance mode) when compared to a microcapillary system

The limited sensitivity obtained in the MCF with colloid nanoparticles in our studies is related to the limited concentration of immobilized nanoparticles that are present in the microfluidic channel during the optical scanning procedure. In the presence of an antigen in the sample, nanoparticles conjugated with the DetAb can be sandwiched to the surface of the microcapillaries pre-coated with CapAb (Fig. 6a), which can form a maximum of 400-ng/cm^2^ monolayer. On the best scenario, nanoparticles form a monolayer around the surface of the microcapillary (Fig. 6b), which is not sufficient to reduce the transmitted light and yield a drop in the grey scale pixel intensity. Note that absorbance values are logarithmically related to the transmitted light, meaning significant absorbance values require a major drop in intensify of transmitted light. Another option in this respect is the use of fluorescence particles.

As compared to the MCF surface, a porous substrate such as a nitrocellulose membrane used in LF tests presents a much larger surface area available for binding the antigen and nanoparticles. Having nominal pore sizes between 0.05 and 12 μm [30] and with antibody-binding capacities of 80–100 μg/cm^2^ [31], nitrocellulose membranes enable the development of assays with a stronger optical signal (Fig. 6c). Therefore, although LF membranes lack sensitivity due to small sample volumes, absence of flow control and autofluorescence properties [32], they present a much higher surface area compared to many microfluidic devices, which is an advantage for particle detection. It should, however, be mentioned that despite a much larger surface area per cm^2^, detection/scanning of coloured nanoparticles used as labels is primarily restricted to the upper part of the nitrocellulose membrane, perhaps down to 5 to 10 μm.

## Conclusion

Although microfluidic systems present a surface area which is very small when compared to LF test membranes, PSA was effectively quantified in the MCF using the silver-enhanced gold nanoparticles, presenting a dynamic range of 10 to 100 ng/ml of PSA. Carbon nanoparticles enable the quantitation of immobilized biotinylated antibody from coating solutions between 10 and 40 μg/ml. The assays used a low-cost flatbed scanner for signal detection. This study shows that optical detection based on nanoparticle immunoassay labelling is possible in the MCF, which opens possibilities for future development of rapid one-step microfluidic immunoassays that can be optically scanned with low-cost optoelectronic components. However, this study clearly highlights that one-step particle detection in microfluidic systems will require major improvements to yield similar sensitivity as in multistep immunoassays.
